# miR-1224-3p Promotes Breast Cancer Cell Proliferation and Migration through PGM5-Mediated Aerobic Glycolysis

**DOI:** 10.1155/2021/5529770

**Published:** 2021-04-19

**Authors:** Fang Ran, Yanan Zhang, Yajiao Shi, Jie Liu, Huayue Li, Lihua Ding, Qinong Ye

**Affiliations:** ^1^Medical School of Guizhou University, Guiyang 550025, China; ^2^Department of Medical Molecular Biology, Beijing Institute of Biotechnology, Beijing 100850, China

## Abstract

Metabolic reprogramming of aerobic glycolysis is a hallmark of cancer cells. Regulators of aerobic glycolysis have become targets for cancer diagnosis and therapy. However, the regulators of aerobic glycolysis in breast cancer development have not been well elucidated. Here, we show that the phosphoglucomutase (PGM) family member PGM5 promotes conversion of glucose-1-phosphate (G1P) into glucose-6-phosphate (G6P) and inhibits breast cancer cell proliferation and migration through regulating aerobic glycolysis. In breast cancer patients, PGM5 is significantly downregulated, and its low expression is a predictor of poor prognosis. MicroRNA-1224-3p (miR-1224-3p) inhibits the PGM5 level through directly targeting its 3'-untranslated region and suppresses PGM5-mediated breast cancer cell proliferation, migration, and glycolytic function. Moreover, the miR-1224-3p/PGM5 axis regulates the expression of cell cycle- and apoptosis-related genes and the markers of epithelial-mesenchymal transition (EMT), a process involved in migration and metastasis of cancer cells. Taken together, our results indicate that miR-1224-3p/PGM5 axis plays important roles in breast cancer cell proliferation, migration, and aerobic glycolysis and may be a potential target for breast cancer therapy.

## 1. Introduction

Breast cancer remains the most common cancer and the leading cause of mortality due to cancer in female around the world [1]. At present, the mortality rate of breast cancer has declined because of the development of early clinical detection methods and improved therapy [2–4]. However, some breast cancer patients have developed advanced breast cancer and have poor prognosis [5]. Therefore, it is crucial to find new targets and improved treatments for breast cancer patients.

Cancer cells have reprogrammed energy metabolism to promote cell proliferation [6–8]. Aerobic glycolysis is a common feature of cancer cells. Even in the presence of sufficient oxygen, cancer cells exhibit increased glycolysis, consume more glucose, and produce more lactic acid, which is called the Warburg effect [9, 10]. In cancer cells, enhanced glycolysis provides more carbon intermediates and precursor molecules for the biosynthesis and other metabolic pathways, thereby promoting anabolic metabolism for the proliferation of cancer cells [6]. Lactic acid produced by aerobic glycolysis alters the cellular microenvironment and promotes the proliferation and migration of cancer cells [11]. The enhancement of glycolysis in cancer cells is predominantly due to the enhanced expression or activity of key glycolytic enzymes [12]. For instance, HK2 (hexokinase 2) and PKM2 (pyruvate kinase M2) are rate-limiting enzymes in the glycolytic pathway [13–15]. HK2 is elevated in many cancers and promotes tumor glycolysis and metastasis [8, 13]. Increased kinase activity of PKM2 is also correlated with poor progression-free survival (PFS) and distant metastatic-free survival (DMFS) in breast cancer patients [16].

PGM5 is a member of phosphoglucomutase (PGM) superfamily, which catalyzes the bidirectional interconversion of glucose-1-phosphate (G1P) and glucose-6-phosphate (G6P), the first intermediate in glycolysis [12, 17, 18]. PGM not only catalyzes sucrose catabolism from G1P to G6P but also catalyzes G6P to G1P, which is a substrate for synthesis of cell wall polymers and glycoproteins [19, 20]. However, the function of PGM5 in glycogen conversion and glycolysis is unknown. Previous studies of PGM5 have focused on its function in muscle tissues [21, 22]. Recent studies indicated that PGM5 is associated with cancer development and progression. PGM5 is downregulated in hepatocellular carcinoma and colorectal cancer [23]. Furthermore, PGM5 inhibits the growth and migration of colorectal cancer cells [24]. These studies suggest that PGM5 may be a tumor suppressor. However, the function of PGM5 in breast cancer has not been reported.

MiRNAs are a family of small noncoding RNAs that block the expression of target genes through translational repression or mRNA degradation. Recently, a wide variety of research studies suggest that microRNAs are glycolysis regulators [25]. However, it remains unclear whether miRNAs directly regulate the expression of PGM5. In this study, we found that miR-1224-3p promoted breast cancer cell proliferation and migration through suppression of PGM5 expression. We found that PGM5 was downregulated in breast cancer patients and inhibited breast cancer cell proliferation, migration, and aerobic glycolysis, accompanied by altered expression of cell cycle- and apoptosis-related genes and EMT markers. Therefore, miR-1224-3p/PGM5 axis in breast cancer may provide new targets for developing antitumor drugs.

## 2. Materials and Methods

### 2.1. Cell Lines, Cell Culture, and Reagents

Human breast cancer cell lines MCF7 and ZR75-1 were obtained from the American Type Culture Collection (ATCC, USA). Cells were cultured in DMEM (Dulbecco's modified Eagle's medium, Invitrogen, Carlsbad, CA, USA) supplemented with 10% fetal bovine serum (Gibco, Carlsbad, CA, USA) and 1% penicillin-streptomycin solution 100X (Corning, CA, USA) in a humidified atmosphere with 5% CO_2_ at 37 °C. Specific antibodies against PGM5 (PA5-48880) was obtained from Invitrogen. Anti-p21 (ab227443), anti-p53 (ab131442), anti-BAX (ab216494), anti-FAK (ab40794), and anti-LDHB (ab112996) were purchased from Abcam. Antibodies against cyclin B (#4138), cyclin D1 (#2922), Bcl-2 (#2876), phos-FAK (Y397) (#8556), MMP-2 (#4022), MMP-9 (#3852), and LDHA (#3582) were obtained from Cell Signaling. Anti-E-cadherin (sc-8426), anti-vimentin (sc-6260), anti-N-cadherin (sc-8424), and anti-*β*-actin (sc-47778HRP) antibodies were purchased from Santa Cruz Biotechnology.

### 2.2. Plasmids Construction and Transfection

The PGM5 ectopic expression vector was constructed by inserting PCR-amplified fragments into pcDNA3 (Invitrogen). PGM5 siRNA and miR-1224-3p mimics and inhibitor were purchased from GenePharma (Suzhou, Jiangsu, China). The sequence of PGM5 siRNA was CCAACUGAAGAUUCGCAUUTT. The sequence of the miRNA-1224-3p inhibitor is AACUAUACAACCUACUACCUCA. The reagent for transfection of plasmids was Lipofectamine 3000 (Invitrogen), and reagent for transfection of miRNA and siRNA was Lipofectamine RNAiMAX (Invitrogen).

### 2.3. Reverse Transcription and Quantitative Real-Time Polymerase Chain Reaction (RT-qPCR)

Total RNA was extracted using TRIzol reagent (Invitrogen, Carlsbad, CA) following the manufacturer's protocol. Equal amount of RNA was reverse transcribed with the oligo primers (the dT primer for PGM5 and actin and the stem-loop primer for miR-1224-3p) using SuperScript II reverse transcriptase (Invitrogen). The stem-loop primer for miR-1224-3p was 5'-GTCGTATCCAGTGCAGGGTCCGAGGTATTCGCACTGGATACGACCTGAGGA-3' and for U6 was 5'-AACGCTTCACGAATTTGCGT-3'. For real-time PCR analysis, the primers for PGM5 were 5'-CTCCTGCATTATCAGGAAG-3' (forward) and 5'-TGTCTTCCTAGTCGAGATAG-3' (reverse). The control primers (*β*-actin) were 5'-GGAAATCGTGCGTGACATT-3' (forward) and 5'-CAGGCAGCTCGTAGCTCTT-3' (reverse). The primers for miR-1224-3p were 5'-CTCGCTTCGGCAGCACA-3' (forward) and 5'-GCAGGGTCCGAGGTATTC-3' (reverse). The control primers (U6) were 5'-CTCGCTTCGGCAGCACA-3' (forward) and 5'-AACGCTTCACGAATTTGCGT-3' (reverse). mRNAs and miRNAs were determined with QuantiFast SYBR Green PCR Kit using the CFX96 real-time PCR detection system. The relative fold expression of the target, normalized to the corresponding control, was calculated by the comparative Ct methods.

### 2.4. Dual-Luciferase Reporter Assay

Wild-type (WT) and mutated (MUT) putative binding sites of miR-1224-3p on PGM5 3'-UTR based on prediction database were cloned into the pmir-GLO dual-luciferase miRNA target expression vector (Promega). The 3'-UTR of human PGM5 gene was amplified by PCR using the following primers: 5'-CCGCTCGAGATAGAGGAAAGATCACTCACC-3' (forward), 5'-ATAAGAATGCGGCCGCTTCTCACTTGGCACAAGTGA-3' (reverse). MUT PGM5 3'-UTR was established by recombinant PCR employing the following primers: 5'-TCTTTCATAGATTACCCTCTATCCCCC-3' (reverse), 5'-GGGGGATAGAGGGTAATCTATGAAAGA-3' (forward). Luciferase reporter assays were performed according to the manufacturer's instructions (Promega). Cells were seeded in 24-well plates and transfected with the WT or MUT PGM5 3'-UTR reporter and miR-1224-3p mimics or negative control using Lipofectamine 3000. The cells were harvested and analyzed for luciferase activities. Firefly luciferase activity was normalized to Renilla luciferase activity as control of transfection efficiency.

### 2.5. Western Blot Analysis

Cells were lysed in RIPA lysis buffer containing protease inhibitors for 30 min. Equal amounts of protein were separated by 10% SDS-PAGE and transferred to a nitrocellulose membrane. The membrane was blocked for 1 h, incubated with indicated antibodies and examined by enhanced chemiluminescence (Promega).

### 2.6. Cell Proliferation

Approximately 3000 cells per well were seeded into 96-well plates and grown for indicated times at 37°C. Cell numbers was determined by CCK-8 Kit (Dojindo Laboratories) according to the manufacturer's protocols. The absorbance at 450 nm of each well was measured by a microplate reader.

### 2.7. Wound Healing Assay

Cells were plated into 6-well plates at the density of 90%. The wounds were scratched via a 200 *μ*L pipette tip to create the wound, followed by washing detached cells with PBS. Then, cells were grown for 16 h. The widths of wounds at 0 h and 16 h were calculated and further examined for cell migration rates.

### 2.8. Lactate Production Assay

Lactate Assay Kit II was used to assess the lactate according to the manufacturer's protocols (BioVision). Cells were plated into 96-well plates at 1000 cells per well and then grown in DMEM containing 10% FBS for 10 h. The media was removed, and the cells were incubated in DMEM without FBS for 1 h. The supernatant was collected for assessment of lactate production.

### 2.9. ATP Assay

To evaluate the ATP production levels of cells, ATP colorimetric assay kit was used to detect the ATP production according to the manufacturer's protocols (BioVision). One million cells were homogenized in 100 *μ*L corresponding assay buffer provided by the kits. Cells were centrifuged, and the soluble fraction was analyzed.

### 2.10. Intracellular Glucose-6-Phosphate (G6P) Assay

Intracellular levels of G6P were measured using a glucose-6-phosphate fluorometric assay kit (Cayman Chemical, #700750) according to the manufacturers' instructions. In brief, cells were harvested and incubated with assay buffer and G6PDH assay reagent for 15 minutes at 37 °C. The absorbance at 590 nm of each well was assessed by a microplate reader.

### 2.11. Statistical Analysis

The statistical analyses were processed by the SPSS 25.0 and GraphPad Prism 7 software. Image J was used to calculate the migration rate. The comparison between the two groups was performed using Student's *t* test. One-way ANOVA was used for comparison among the different groups. Survival analysis was performed using the Kaplan–Meier test. *P* values of <0.05 were considered statistically significant. All the experiments in vitro were performed in triplicate and repeated 3 times.

## 3. Results

### 3.1. PGM5 Expression Is Downregulated in Breast Cancer Tissues

**Figure 1:**
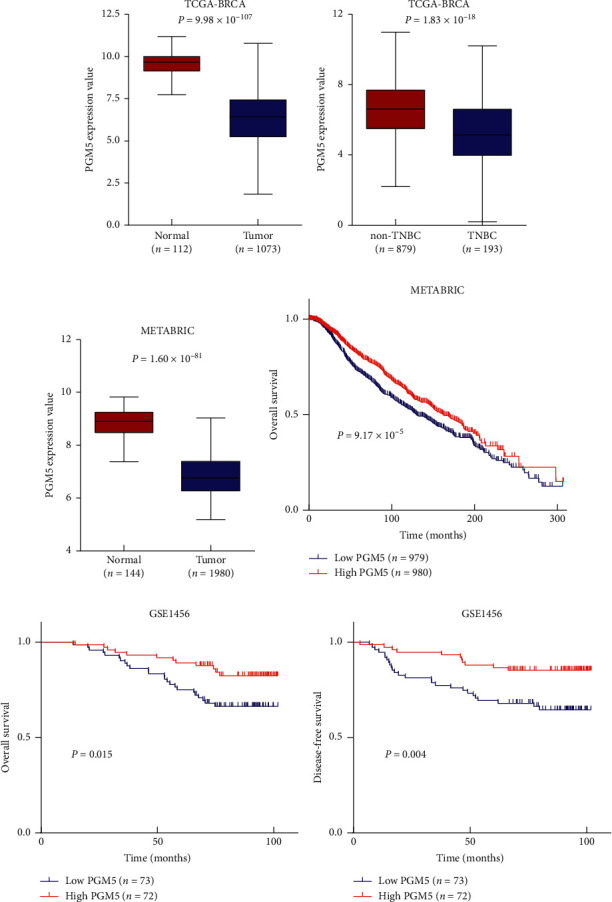
PGM5 expression is downregulated in breast cancer tissues. (a–c) PGM5 expression values were plotted and compared between 112 adjacent normal and 1073 tumor tissues based on the TCGA-BRCA database (a), 879 non-TNBC and 193 TNBC tissues based on the TCGA-BRCA database (b), and 144 normal and 1980 tumor tissues based on the METABRIC database (c) with the Mann–Whitney *U* test. (d) Kaplan–Meier survival curves for overall survival of 1959 patients with follow-up information according to the relative expression of PGM5 from the METABRIC dataset. (e, f) Kaplan–Meier survival curves for overall survival (e) and disease-free survival (f) of 145 patients in the GSE1456 database.

To detect the function of PGM5 in cancer, we first investigated the clinical significance of PGM5 in breast cancer. The level of PGM5 was decreased in breast cancer patients compared to the adjacent nontumor normal tissues in the Cancer Genome Atlas (TCGA) breast cancer (BRCA) database (Figure 1(a)). Interestingly, the PGM5 expression in triple-negative breast cancer (TNBC) patients was lower than that in non-TNBC patients (Figure 1(b)). Moreover, PGM5 was also significantly downregulated in the Molecular Taxonomy of Breast Cancer International Consortium (METABRIC) database (Figure 1(c)). Furthermore, patients with high PGM5 expression had longer overall survival (OS) according to the METABRIC and GSE1456 databases and had longer disease-free survival (DFS) based on GSE1456 (Figures 1(d)–1(f)). Taken together, these results suggest that PGM5 is a good prognostic factor in breast cancer patients.

### 3.2. PGM5 Suppresses Proliferation and Migration of Breast Cancer Cells

**Figure 2:**
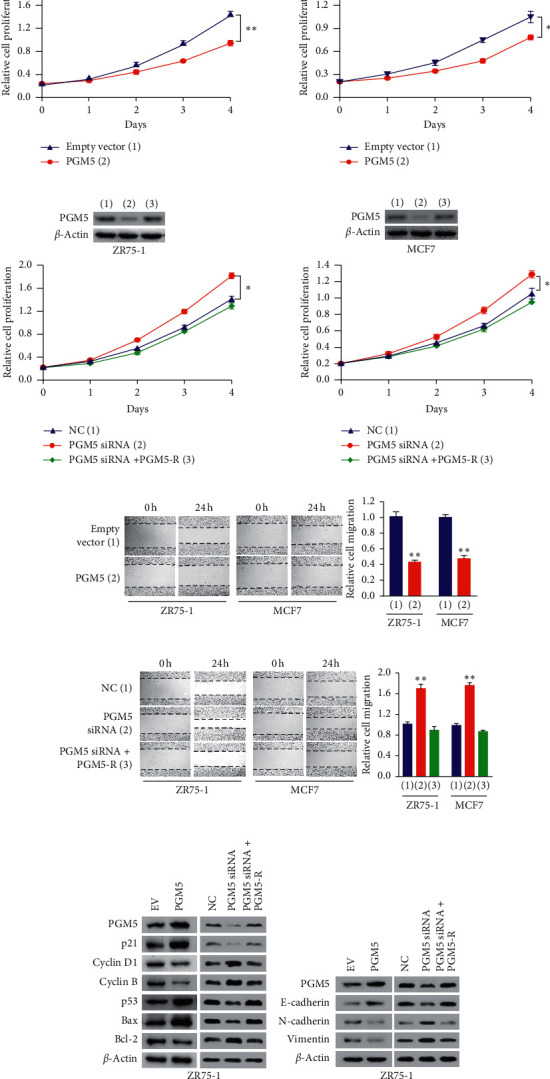
PGM5 suppresses proliferation and migration of breast cancer cells. (a) Cell proliferation assays of ZR75-1 (left panel) and MCF7 cells (right panel) transfected with the empty vector or PGM5. PGM5 expression was examined by immunoblot with actin as a loading control. (b) Cell proliferation assays of ZR75-1 (left panel) and MCF7 cells (right panel) transfected with control siRNA, PGM5 siRNA, or PGM5 siRNA plus siRNA-resistant PGM5 expression vector (PGM5-R). PGM5 expression was detected by immunoblot. (c, d) Wound healing assays of ZR75-1 and MCF7 cells transfected as in (a) and (b), respectively. The values of the control group in ZR75-1 and MCF7 cells were set to 1. (e, f) Immunoblot analysis of ZR75-1 cells transfected with the empty vector or PGM5 or control siRNA, PGM5 siRNA, or PGM5 siRNA plus PGM5-R with the indicated antibodies. Data shown are mean ± SD of triplicate measurements that were repeated three times with similar results (a–d). ^*∗*^*P* < 0.05; ^*∗∗*^*P* < 0.01.

Next, we detected the effect of PGM5 on proliferation and migration of breast cancer cells. Cell proliferation assay showed that ZR75-1 and MCF7 cells transfected with PGM5 grew more slowly than those with the empty vector (Figure 2(a)). In contrast, transient knockdown of PGM5 increased proliferation of these cells (Figure 2(b)). These effects could be rescued by PGM5 reexpression in the PGM5 knockdown cells. Wound healing assay showed that overexpression of PGM5 inhibited migration capability of ZR75-1 and MCF7 cells (Figure 2(c)). PGM5 knockdown in these cells promoted their migration capability, and reexpression of PGM5 in the knockdown cells rescued these effects (Figure 2(d)). These data suggest that PGM5 plays an important role in proliferation and migration of breast cancer cells.

Since cell cycle and apoptosis are involved in cell proliferation, we tested the effect of PGM5 on expression of cell cycle- and apoptosis-related genes in breast cancer cells. Western blot assay showed that overexpression of PGM5 increased p21 expression and decreased cyclin B and cyclin D1 expression, while knockdown of PGM5 decreased p21 expression and promoted cyclin B and cyclin D1 expression (Figure 2(e)). Reexpression of PGM5 in the knockdown cells rescued these effects. As far as apoptosis-related genes are concerned, PGM5 overexpression promoted p53 and Bax expression and inhibited Bcl-2 expression (Figure 2(e)). Consistently, PGM5 knockdown showed the opposite effects, and reexpression of PGM5 in the knockdown cells rescued these effects.

Epithelial-mesenchymal transition (EMT) was shown to play a critical role in cancer cell migration, so we detected if PGM5 also regulates EMT of breast cancer cells. Western blot showed that overexpression of PGM5 increased expression of E-cadherin, the epithelial marker, and decreased expression of N-cadherin and vimentin, two mesenchymal markers (Figure 2(f)). Knockdown of PGM5 reduced expression of E-cadherin and promoted expression of N-cadherin and vimentin, and reexpression of PGM5 in the knockdown cells rescued these effects. These data suggest that PGM5 may suppress EMT.

### 3.3. miR-1224-3p Inhibits PGM5 Expression by Directly Targeting Its 3'-UTR

**Figure 3:**
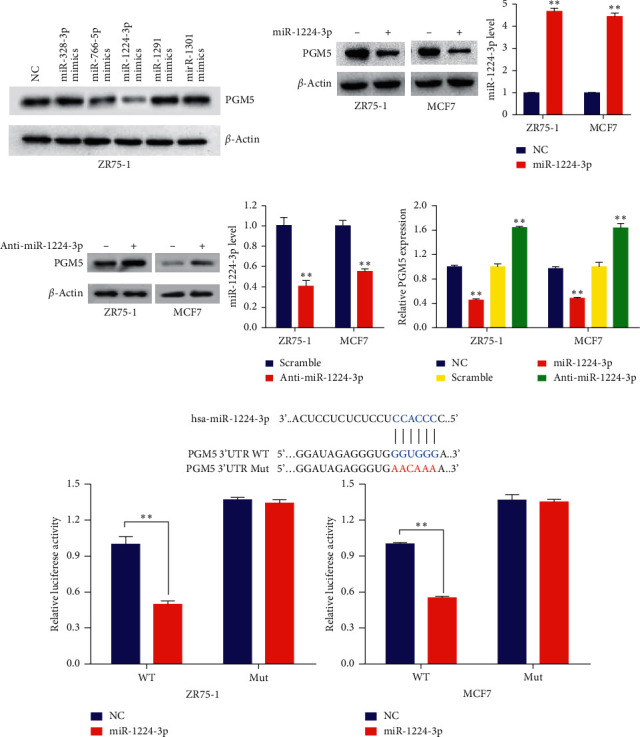
miR-1224-3p inhibits PGM5 expression by directly targeting its 3'-UTR. (a) ZR75-1 cells were transfected with NC (negative control) or mimics of candidate miRNAs as indicated. PGM5 expression was tested by immunoblot. (b, c) ZR75-1 and MCF7 cells were transfected with (b) NC or miR-1224-3p mimics and (c) scramble control or miR-1224-3p inhibitor (anti-miR-1224-3p). Histograms show relative miR-1224-3p expression by RT-qPCR. (d) RT-qPCR analysis of PGM5 mRNA expression in ZR75-1 and MCF7 cells transfected as in (b) and (c). (e) miRNA luciferase reporter assays in ZR75-1 and MCF7 cells transfected with wild-type (WT) or mutated (MUT) PGM5 reporter plus miR-1224-3p mimics. The top panel indicates WT and MUT forms of putative miR-1224-3p target sequences of PGM5 3'-UTR. Blue font indicates the putative miR-1224-3p binding sites within human PGM5 3'-UTR. Red font indicates the mutations introduced into the PGM5 3'-UTR. Data shown are mean ± SD of triplicate measurements that were repeated three times with similar results. ^*∗∗*^*P* < 0.01.

We used two target prediction tools, TargetScan and miRanda database, to screen the potential miRNAs targeting PGM5. Five potential miRNAs were identified, including miR-328-3p, miR-766-5p, miR-1224-3p, miR-1291, and miR-1301. Western blot analysis in ZR75-1 cells showed that miR-1224-3p greatly reduced PGM5 expression, miR-766-5p had a mild effect, and the other three had no effect (Figure 3(a)). Thus, we chose miR-1224-3p for further study. miR-1224-3p mimics suppressed PGM5 expression in ZR75-1 and MCF7 cells (Figure 3(b)). In contrast, the miR-1224-3p inhibitor promoted PGM5 expression in these cells (Figure 3(c)). Moreover, qRT-PCR analysis showed that miR-1224-3p mimics decreased the PGM5 mRNA level, whereas the miR-1224-3p inhibitor increased PGM5 mRNA expression in ZR75-1 and MCF7 cells (Figure 3(d)).

To detect whether miR-1224-3p directly targets the PGM5 3'-UTR region, we performed luciferase reporter assay with PGM5 wild-type or mutated 3'-UTR luciferase reporters and miR-1224-3p mimics in ZR75-1 and MCF7 cells. The results indicated that miR-1224-3p mimics inhibited the luciferase reporter activity of wild-type 3'-UTR, but not the mutated 3'-UTR (Figure 3(e)). Therefore, these results suggest that miR-1224-3p suppresses PGM5 expression through directly targeting its 3'-UTR.

### 3.4. miR-1224-3p Promotes Cell Proliferation and Migration through Inhibiting PGM5 Expression

**Figure 4:**
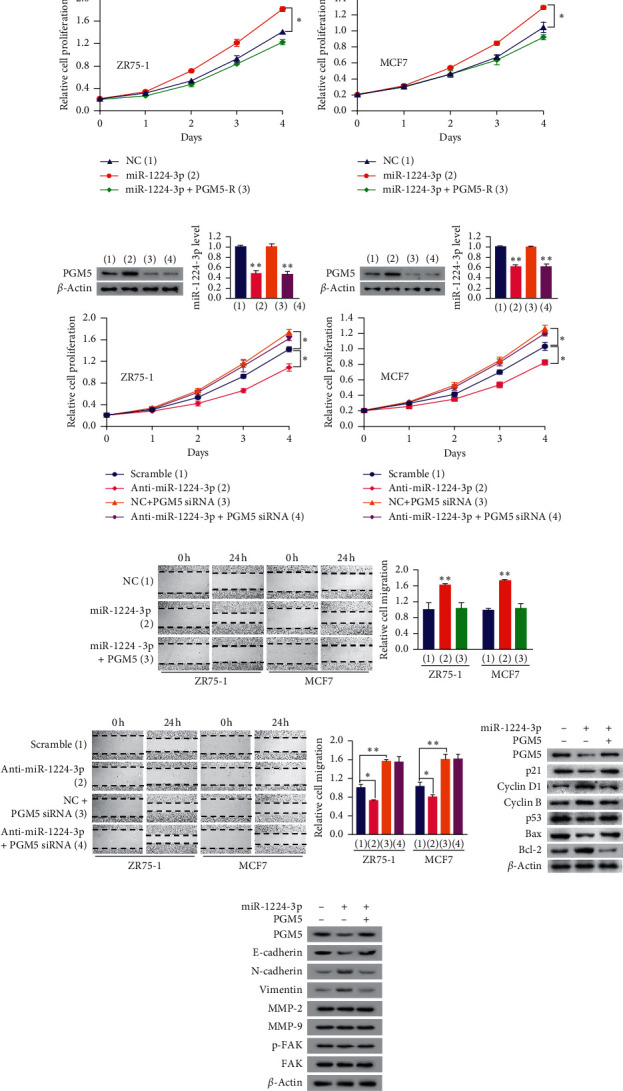
miR-1224-3p promotes cell proliferation and migration through inhibiting PGM5 expression. (a, b) Cell proliferation assays of ZR75-1 cells (left panel) or MCF7 cells (right panel) transfected with (a) miR-1224-3p mimics or miR-1224-3p mimics plus PGM5 expression plasmid or (b) anti-miR-1224-3p, PGM5 siRNA, or anti-miR-1224-3p plus PGM5 siRNA as indicated. Immunoblot analysis shows PGM5 expression. RT-qPCR shows miR-1224-3p expression. (c, d) Wound healing assays of ZR75-1 and MCF7 cells transfected as in (a) and (b). Histograms show relative cell migration. (e, f) Immunoblot analysis of ZR75-1 cells transfected as in (a). Data shown are mean ± SD of triplicate measurements that were repeated three times with similar results (a–d). ^*∗*^*P* < 0.05; ^*∗∗*^*P* < 0.01.

Next, we investigated the biological function of miR-1224-3p in breast cancer cells and tested whether miR-1224-3p exhibits these functions through PGM5. Compared with the control group, miR-1224-3p mimics significantly increased the proliferation and migration of ZR75-1 and MCF7 cells (Figures 4(a) and 4(c)). These effects could be rescued by PGM5 reexpression in the miR-1224-3p-transfected cells. In contrast, miR-1224-3p inhibitor suppressed the proliferation and migration of these cells (Figures 4(b) and 4(d)). Importantly, PGM5 knockdown abolished the ability of miR-1224-3p inhibitor to decrease proliferation and migration of breast cancer cells. Moreover, miR-1224-3p mimics enhanced the expression of the cell cycle-related genes cyclin B and cyclin D1, the apoptosis-related gene Bcl-2, and the migration-related genes N-cadherin and vimentin and decreased the expression of the cell cycle-related genes p21 and p53, the apoptosis-related gene Bax, and the migration-related gene E-cadherin (Figures 4(e) and 4(f)). Reexpression of PGM5 in the miR-1224-3p-transfected cells rescued these effects. However, miR-1224-3p mimics did not alter the expression of the motility/metastasis-related genes MMP-2, MMP-9, and FAK, as well as phosphorylated FAK (p-FAK), and reexpression of PGM5 in the miR-1224-3p-transfected cells did not have an effect on expression of these genes (Figure 4(f)). Taken together, these findings indicate that miR-1224-3p mediates the proliferation and migration of breast cancer cells through PGM5.

### 3.5. miR-1224-3p/PGM5 Axis Modulates Glycolysis in Breast Cancer Cells

**Figure 5:**
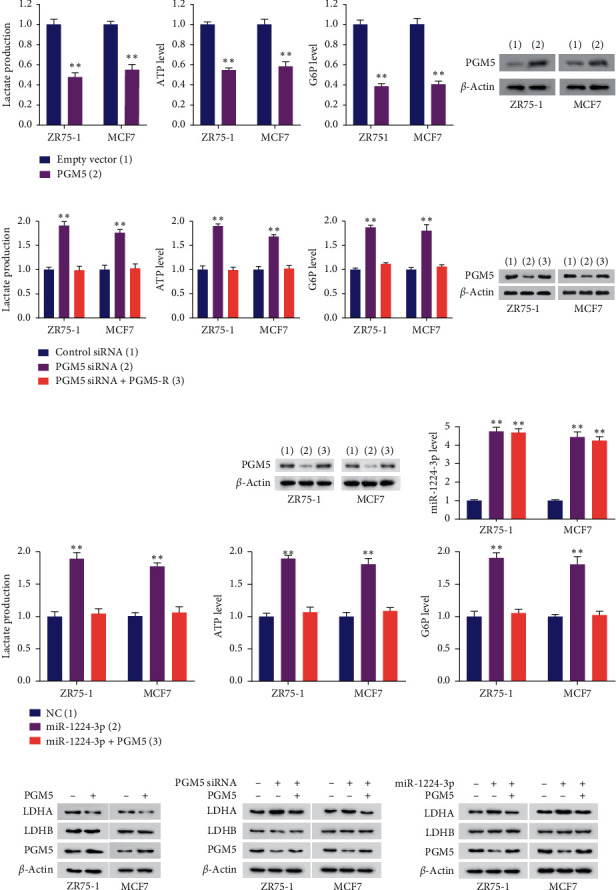
The miR-1224-3p/PGM5 axis modulates glycolysis in breast cancer cells. (a, b) The production of lactate, ATP, and G6P was determined in ZR75-1 and MCF7 cells transfected with (a) empty vector or PGM5 or (b) NC, PGM5 siRNA, or PGM5 siRNA plus PGM5-R. Immunoblot analysis indicates PGM5 expression. (c) The production of lactate, ATP, and G6P was detected in ZR75-1 and MCF7 cells transfected with miR-1224-3p or miR-1224-3p plus PGM5-expressing plasmid. Representative immunoblot reveals PGM5 expression. RT-qPCR indicates miR-1224-3p expression. (d) ZR75-1 and MCF7 cells were transfected as in (a) or (b). Expression of LDHA, LDHB, and PGM5 was detected by immunoblot analysis. (e) Immunoblot analysis of ZR75-1 and MCF7 cells transfected as in (c). Data shown are mean ± SD of quintuplicate measurements that were repeated three times with similar results. Data shown are mean ± SD of triplicate measurements that were repeated three times with similar results (c for RT-qPCR analysis). ^*∗∗*^*P* < 0.01.

Since PGM is a key enzyme in the metabolism of glucose-1-phosphate and glucose-6-phosphate, we detected the function of miR-1224-3p/PGM5 axis on aerobic glycolysis. Overexpression of PGM5 decreased production of lactate, ATP, and G6P in ZR75-1 and MCF7 cells (Figure 5(a)). Oppositely, PGM5 knockdown promoted production of lactate, ATP, and G6P in these cells, suggesting PGM5 regulates glycolysis and conversion of G1P to G6P (Figure 5(b)). Similar to PGM5 knockdown, miR-1224-3p mimics increased production of lactate, ATP, and G6P (Figure 5(c)). These effects could be reversed by reexpression of PGM5 in the miR-1224-3p-transfected cells. Due to the alteration of lactate production, we further determined the effect of the miR-1224-3p/PGM5 axis on LDH expression. The results showed that overexpression of PGM5 inhibited expression of LDHA, but not LDHB, and knockdown of PGM5 increased expression of LDHA, but not LDHB (Figure 5(d)). Reexpression of PGM5 in the PGM5 knockdown cells rescued the effect of PGM5 knockdown on LDHA expression. Moreover, miR-1224-3p promoted expression of LDHA, but not LDHB, and reexpression of PGM5 in the miR-1224-3p-transfected cells reversed the effect of miR-1224-3p on LDHA expression (Figure 5(e)). These results suggest that miR-1224-3p/PGM5 axis regulates aerobic glycolysis in breast cancer cells.

### 3.6. miR-1224-3p/PGM5 Axis Modulates Breast Cancer Cell Proliferation and Migration through Aerobic Glycolysis

**Figure 6:**
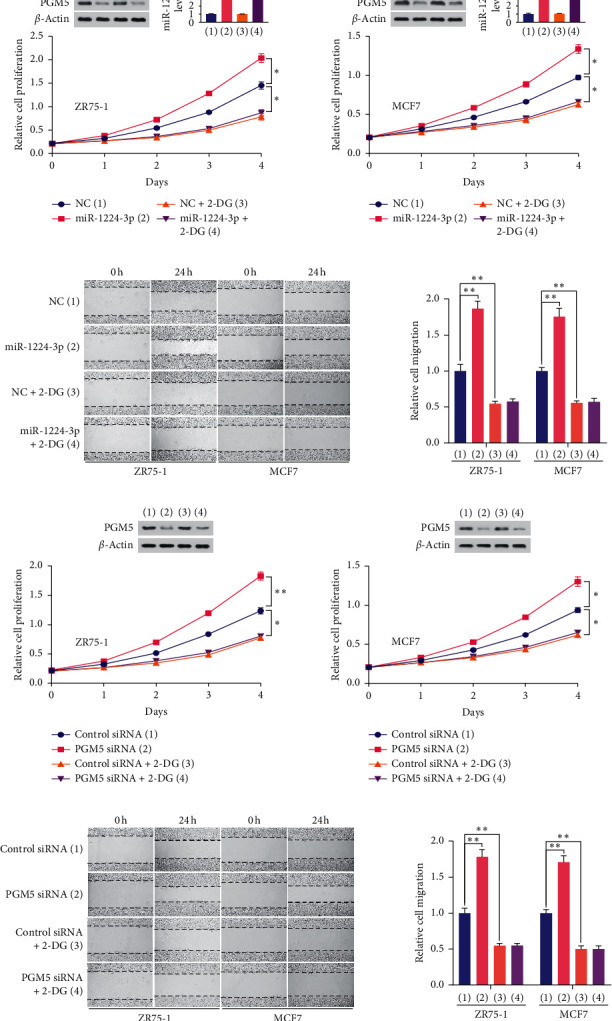
The miR-1224-3p/PGM5 axis modulates breast cancer cell proliferation and migration through aerobic glycolysis. (a) The proliferation curve of ZR75-1 and MCF7 cells transfected with miR-1224-3p or scramble and treated with 2.5 mM 2-DG as indicated. Representative immunoblot reveals the expression of PGM5. RT-qPCR indicates miR-1224-3p expression. (b) Wound healing assays of ZR75-1 and MCF7 cells transfected as in (a). (c) The proliferation curve of ZR75-1 and MCF7 cells transfected with NC or PGM5 siRNA and treated with 2.5 mM 2-DG as indicated. Representative immunoblot reveals the expression of PGM5. (d) Wound healing assays of ZR75-1 and MCF7 cells transfected as in (c). Histograms show relative cell migration. All values shown are mean ± SD of triplicate measurements and have been repeated 3 times with similar results. ^*∗*^*P* < 0.05; ^*∗∗*^*P* < 0.01.

Next, we investigated whether miR-1224-3p/PGM5 axis regulates breast cancer cell proliferation, migration, and invasion through glycolysis. With cell proliferation assay, wound healing assay, and transwell assay, we found that glycolytic inhibitor 2-deoxy-D-glucose (2-DG) abolished miR-1224-3p- and PGM5 knockdown-mediated promotion of proliferation and migration in breast cancer cells (Figures 6(a)–6(d)), suggesting that the miR-1224-3p/PGM5 axis regulates breast cancer cell proliferation and migration through glycolysis.

## 4. Discussion

The reprogrammed glycolysis is a hallmark of cancer and is closely associated with prognosis of breast cancer patients [6]. Since cancer cells favor glycolysis as an energy supply, glycolysis has become targets for cancer treatment [26–28]. The interference of glycolysis modulators could inhibit breast cancer development and progression. Therefore, the investigation of glycolysis modulators provides targets for breast cancer therapy.

PGM5 has been found to play a role in cancer. However, how PGM5 expression is regulated, and whether PGM5 regulates glycolysis and breast cancer development and progression remains unclear. In the current study, we found important roles for PGM5 in breast cancer cell glycolysis, proliferation, and migration, as well as prognosis of breast cancer patients. First, we found that PGM5 inhibited the conversion of G1P to G6P. Second, the level of PGM5 is significantly downregulated in breast cancer patients compared to normal breast tissue, and the expression of PGM5 is positively correlated with DFS and OS. Third, we identified miR-1224-3p to be a novel inhibitor of PGM5. Fourth, miR-1224-3p/PGM5 axis promotes breast cancer proliferation and migration and regulates expression of cell cycle- and apoptosis-related genes and EMT markers. Fifth, we found that miR-1224-3p enhances breast cancer cell proliferation and migration through glycolysis. These findings demonstrated that miR-1224-3p/PGM5 axis may be a therapeutic target for breast cancer patients.

PGM has been found to be associated with proliferation, migration, and metastasis of cancer [12, 17, 18, 24]. However, the mechanism of PGM family regulating cancer progression has not been investigated. In the current study, we found a critical role of PGM5 in modulating cell cycle- and apoptosis-related gene expression. PGM5 significantly reduced the expression of the G1/S phase marker cyclin D1 but increased the level of p21, the major regulators of G1/S phase transition [29, 30]. Meanwhile, PGM5 inhibited the expression of the antiapoptotic protein Bcl-2 and promoted the expression of the apoptotic proteins BAX [31]. Importantly, miR-1224-3p inhibited PGM5-mediated regulation of cell cycle and apoptosis modulators. We also showed for the first time that PGM5 regulates the expression of EMT markers, suggesting that PGM5 may regulate EMT.

The role of miR-1224-3p in cancer is rarely known. miR-1224-3p has been reported to target ETV1 in lung adenocarcinoma cells. miR-1224-3p suppresses circZNF609-ETV1 axis-induced malignant phenotype [32]. miR-1224-3p is a sensitive marker for bladder cancer detection [33]. However, the function of miR-1224-3p in glycolysis and breast cancer has not been investigated. In the present study, we demonstrated that miR-1224-3p enhanced glycolysis, breast cancer cell proliferation, and migration through targeting PGM5.

In summary, this is the first study to identify the glycolytic function of PGM5. miR-1224-3p directly reduces the expression of PGM5 to suppress proliferation and migration through glucose reprogramming. Our results suggest the important roles of the miR-1224-3p/PGM5 axis in regulating glycolysis and breast cancer growth and progression. Repression of miR-1224-3p or activation of PGM5 may be useful strategies for breast cancer therapy.

## Data Availability

The data used to support the findings of this study are available from the corresponding author upon request.
